# The Research on Risk Factors for Adolescents’ Mental Health

**DOI:** 10.3390/bs14040263

**Published:** 2024-03-22

**Authors:** Jiayu Lin, Wuyuan Guo

**Affiliations:** 1 Department of Psychology, University of Michigan, Ann Arbor, MI 48109, USA; 2 Department of Curriculum and Instruction, The Education University of Hong Kong, Hong Kong 999077, China; s1124027@alumni.eduhk.hk

**Keywords:** adolescents, mental health, depression, self-efficacy, parenting styles

## Abstract

There is a growing tendency for mental health disorders to emerge during adolescence. These disorders impair emotional, cognitive, and behavioral functioning, such as unsatisfying peer relationships, disruptive behavior, and decreased academic performance. They also contribute to vulnerability in later adulthood which negatively influences life-long well-being. Thus, research into etiology is imperative to provide implications for prevention and intervention within family and school practices. It is suggested that the onset of psychological disorders, such as depression and anxiety, is closely related to stress levels and patterns of stress reaction. Therefore, considerable research has investigated the link between hereditary factors, economic status, dispositional vulnerability, social relationships, and stress levels. The current study examines existing evidence and identifies multifaceted risk factors for adolescents’ mental problems across three layers, including individual traits and personality, family status and practices, as well as peer relationships, and school climate. It is also suggested that factors from these three perspectives interact and are closely interconnected, directly or indirectly contributing to adolescent psychopathology. The implications for future development of prevention and intervention programs, as well as therapy, are discussed.

## 1. Introduction

The mental health issues of youth have received great concern from psychologists and educators because of their life-span influence on healthy functioning and well-being. Worldwide, one out of every seven youths aged 10 to 19 years old experience a mental disorder [1]. Depression, anxiety, and behavioral disorders are the primary indicators of impairment in teenagers [1]. Psychiatric epidemiological surveys conducted globally have found a prevalence of 17.5% to 19.9% for any psychiatric disorder among adolescents [2,3]. From 2009 to 2019, there has been a notable rise in the prevalence of sadness and hopelessness among high school students, rising from 26.1% to 36.7% [4]. Additionally, there has been an increase in the incidence of suicide planning and attempted suicide [4]. Adolescence is a critical phase in which the foundations for health, cognitive, and emotional abilities are established, and the influence of health trajectory extends throughout life [5]. From the perspective of social and economic development, mental illnesses developed in childhood cost more than those in adulthood in aspects of healthcare expenses, special education demands, the burden on the criminal justice system, and social welfare programs [6].

Individual characteristics and traits have been explored in previous research to be associated with mental health outcomes. Self-esteem and self-efficacy, for example, involve the cognitive appraisal and evaluation of self-capability and self-image. Based on the social cognitive theory, the perceived inability to act gives rise to anxiety, a sense of futility, and depressive states [7]. Later studies on adolescents confirmed this claim, finding a relationship between self-inefficacy, depressive symptoms, and anxiety disorders [8]. Cognitive functions are bidirectionally related to mental health problems, as they act both as risk factors for and consequences of mental illnesses. Research indicated a bidirectional relationship of self-esteem on depression, anxiety, as well as well-being among adolescent students [9]. The maladaptive perfectionism trait is also considered as a potential risk factor triggering depression, anxiety, and decreased mental health in teenagers [10].

Previous research has explored various factors associated with psychiatric disorders in youth. The family environment and parenting practices play an essential role in children’s physical and psychological development as well as their behavioral patterns. Substantial evidence has suggested the robust associations between parental socioeconomic status, mental health status, family dynamics (i.e., the way family members interact), and children’s mental health problems [11,12]. Over a six-year period, a longitudinal study revealed a bidirectional and transactional influence between parenting styles and children’s ADHD, ODD, depression, and anxiety symptoms [13]. Among 921 junior high school students in China, a study suggested that family dysfunction is a predictor of psychopathology (i.e., anxiety and depression) [14].

In addition to family members, adolescents spend most of their time in school with their peers, and both peer relationships and group climate have a significant impact on their emotional health and well-being. In peer relationships outside the family, victimizing bullying accounts for the prominent causative factor leading to poor general psychological health outcomes including depression syndromes, anxiety, and suicidal ideation and attempts [15]. Instead of viewing teens’ victimization as a phenomenon between two individuals, the social culture or, more specifically, school climate should be considered in research and practice. A feeling of school safety and better teacher–student and peer relationships are associated with improved affective, behavioral, and cognitive outcomes, school attachment, and academic performance [16,17,18]. Besides traditional peer connection, virtual communication featured by media use is prevalent among adolescents in the information age. However, the relevant research indicated mixed effects of adolescents’ social media use. A general ameliorating effect of social media use on mental problems was concluded in a systematic review [19], but another review study suggested a negative relationship between social media use and depression [20].

Although previous research has explored how family and social relationships are related to teenagers’ mental health, few studies involved cognitive function and linked these factors together. Therefore, the current study conducted a comprehensive review of the empirical evidence on how youth mental health and well-being are affected by individual cognitive characteristics, family environment, and social relationships. The purpose of this research is to synthesize updated research findings of the relevant fields and provide in-depth analysis of pathways through which multifaceted risk factors lead to mental health problems in teenagers. The current study is warranted due to its potential to inspire and serve as a reference for policymaking, social services, school education, and interventions targeted at improving adolescents’ positive functioning.

## 2. Method

There are a variety of risk factors associated with adolescents’ psychopathology. Extensive research has focused on the detailed mechanism of a single aspect of disorder development, yet there is a lack of comprehensive studies summarizing these findings from various perspectives. In this study, we aim to identify factors across three levels, individual, family, and broader social relationships, to capture their influences on general well-being and specific mental disorders. 

### 2.1. Searching Strategies

A total of 35 empirical articles are included in this review. Two databases were utilized for the search: PsycINFO and Google Scholar. Articles were mainly search by keywords and their derivatives, including “adolescents”, “mental health”, “depression”, “anxiety”, “self-esteem”, “self-esteem”, “self-efficacy”, “perfectionism”, “parenting styles”, “family practices”, “ACEs”, “peer victimization”, “peer supports”, “peer rejection”, and “school climate”. In addition to searching from databases, extra studies were drawn from the references lists of critical systematic review studies.

### 2.2. Inclusion, Exclusion, and Classification Criteria

In order to present studies that are updated, relevant, and of high quality, the following inclusion and exclusion criteria were established. 

Inclusion criteria: (1) Studies exploring potential factors affecting mental health outcomes and the development of mental disorders. (2) The target population of the majority of studies included should include children and adolescents aged 5–18. There is one exception for a study with participants aged below 5, because of implications for prospective and continuous influences on adolescents. (3) The time frame of experimental studies is limited to the 21st century onwards. (4) The samples drawn in the studies should be representative, with relatively large sample sizes. (5) Studies with relatively high citation rates and impact factors.

Exclusion criteria: (1) Not in English. (2) Lack of reliable measurements. (3) Lack of scientific statistical analysis. (4) Not original findings.

Classification criteria: (1) Classified according to the main themes: individual-related, family/parent-related, and social relationship-related. (2) Classified further into subtopics under each main theme according to the constructs investigated in the study.

## 3. Individual Characteristics

Table 1 summarizes papers reviewed for the association between individual vulnerabilities and mental health outcomes. Eleven cross-sectional and longitudinal studies are involved covering self-esteem, self-efficacy, and perfectionism.

### 3.1. Self-Esteem

Self-esteem is an essential construct related to the perception of the self. It refers to one’s overall attitude and evaluation towards the self which represents self-concepts and self-worth [29]. An evaluation of the self is formed through inner reflection or comparison to the outer world. Failure to meet self-imposed standards or facing rejection by others could both contribute to a decrease in one’s self-esteem. As proposed by the vulnerability model, lower self-esteem plays a powerful causal role in the development of depression [30]. The model has been further strengthened by controlling other relevant personality factors [31]. 

Due to the habitual attribution style formed by past experiences, adolescents with low self-esteem are predisposed to experiencing more negative affect and gloomy moods. Also, adolescents with lower self-esteem tend to underestimate their abilities when encountering difficulties and more frequently feel frustrated. Being immersed in the adverse appraisal of self leads to more depressive symptoms and even prolonged depression. The predictiveness of self-esteem on depression was tested among 351 adolescents at school [9]. The results revealed that teenagers who scored lower on self-esteem were more likely to have higher levels of depression, anxiety, and a lower level of overall mental well-being [9]. The study supported the vulnerability model of self-esteem and depression in which negative self-evaluation and coping resources lead to elevated maladjustment when individuals face stressful life events [9]. Longitudinal studies have also supported the vulnerability model of self-esteem and depression. In samples of junior high school students with an average age of 12.7, self-report surveys were conducted five times within two years [21]. It was found that self-esteem negatively predicts adolescents’ common mental health problems such as depression, anxiety, emotional instability, maladjustment, obsessive–compulsive symptoms, and paranoia [21]. 

### 3.2. Self-Efficacy

Self-efficacy is closely linked to mental health outcomes for adolescents. It is relevant to the connection between an individual’s cognitive depreciation on the self and the behavioral coping inability when faced with challenges [32]. A body of empirical research demonstrates that lower self-efficacy related to academic ability and social relationships is associated with more depressive symptoms in adolescents [22,23], while higher self-efficacy is associated with reduced externalizing problems [25]. Self-efficacy plays a crucial role as an essential mediator in influencing mental health outcomes. A study with samples from different cultures (Germany, Russia, and China) demonstrated its mediating role in the relationship between daily stress and depression, anxiety, and multi-aspect psychosocial well-being [24]. Self-efficacy was found to mediate the protective role of growth mindset and persistence in anxiety, depression, and externalizing problems, although with gender differences [25].

Self-efficacy plays a critical role in the etiological framework of depression. Rhem proposed that the depression manifests as a deficiency in the self-control system that involves the processes of self-evaluation, self-monitoring, and self-reinforcement [33]. Self-efficacy exerts a noticeable influence on the diverse dimensions of the self-control process. Notably, adolescents with low self-efficacy may attribute unfavorable outcomes to their inner limitations rather than to external factors. Furthermore, they may lack confidence in achieving their goals and tend to set up high standards for appraising themselves.

Self-efficacy is also a reliable predictor of one’s behavior in terms of the effort invested in problem solving and perseverance when facing challenges in adverse situations [34,35]. Low self-efficacy increases the likelihood of forming negative preconceptions about one’s capacities. These preconceptions of inadequacy can interfere with problem solving and task completion, negatively impacting various aspects of teenagers’ lives, including goal achievement, academic performance, and social network building. According to the diathesis–stress model [36], it is the stressor coming from the environment that accumulates to a certain level that triggers the onset of mental illness. Negative pre-assumptions add to the stress level from the beginning of problem solving and can further elevate stress levels when frustration is not successfully solved. A cross-sectional study reported that domain-specific self-efficacy is related to domain-specific anxiety among adolescents [8]. Social self-efficacy was connected with social phobia, while emotional self-efficacy was connected to panic disorder and generalized anxiety [8]. When the neuroticism trait was controlled, self-efficacy still significantly predicted depression and anxiety disorder in adolescents [8]. Therefore, teenagers with low self-efficacy are more vulnerable to developing mental disorders.

### 3.3. Perfectionism

Perfectionism is a dispositional trait characterized by the tendency to strive for excessively high goals, often leading to self-blame due to the discrepancy between the actual performance and the ideal standard. As it is closely related to academic goals and peer interactions which are major aspects of students’ lives, adolescents who strive for unrealistic achievements are more vulnerable to mental health problems. A review study indicated maladaptive perfectionism, as an underlying mechanism, might serve as an underlying mechanism leading to the development of depression and anxiety in the pediatric population [37]. Perfectionism can be categorized into three types as shown in Table 2. The multidimensional construct conceptualized three dimensions of perfectionism: self-oriented perfectionism, other-oriented perfectionism, and socially prescribed perfectionism [38]. A meta-analysis that included 121 studies with 41,824 participants aged 7–24 showed that perfectionistic concerns exhibited medium pooled correlations with symptoms of anxiety, obsessive–compulsive disorder (OCD), test anxiety, and depression among young people, while perfectionistic strivings demonstrated smaller yet still significant associations with symptoms of anxiety and OCD [39]. Perfectionist adolescents are afraid of failure and weakness, as it threatens their perfect image in the eyes of others. They may be reluctant to seek assistance when they actually need it, because they tend to uphold a positive self-image which appears to stem from perfectionistic style of self-presentation [40]. As a result, their coping resources plummet when they are confronted with problematic situations. Coupled with diminishing support from others, anxiety arises as they worry about whether their performances will meet the standard of perfection.

The detrimental impact of perfectionism on adolescents’ mental health is displayed through self-criticism, self-punishment, and the establishment of rigorous standards, which are typical features of self-oriented perfectionism [38]. A longitudinal examination of self-critical perfectionism and mental health in a sample of high school students in Canada reveled a positive association between perfectionism and depressive and anxiety symptoms [10]. Another study suggested that students who exhibited higher levels of self-critical perfectionism experienced more severe anxiety and depression before starting university, with these symptoms either intensifying or remaining consistent over time, unlike their counterparts with lower levels of self-critical perfectionism [26]. Unrealistically high standards are often contradicted by reality, particularly due to environmental factors outside individual control. Although the high standards of self-oriented perfectionists stem from internal motivation, failing to meet these standards frequently results in self-blame and self-doubt on their competence and decision making. This disappointment, combined with a ruminative cognitive style, leads to constant stress and negative emotions. Ultimately, it may cause psychological disorders in perfectionist adolescents.

In contrast to self-oriented perfectionism, other-oriented perfectionism is directed outward, characterized by placing unrealistic requirements and conducting stringent evaluations on the performance of significant others [38]. There is an internal assumption that others have a duty to strive for perfection, leading other-oriented perfectionists to be highly critical towards others regarding imperfections [27]. The style of idealized cognitive operation endangers mental health by harming adolescents’ ability to form satisfactory interpersonal relationships with family members, friends, and peers at school. Youths who impose perfect standards on others tend to be more hostile and blame others for undesired performances, leading to interpersonal frustration. Research has demonstrated unique links between other-oriented perfectionism and the dark triad of personalities: narcissism, Machiavellianism, and (subclinical) psychopathy [27]. Although less attention has been paid to the direct effect of other-oriented perfectionism on mental health outcomes, it could be implied that without positive interpersonal relationships, adolescents are likely to lack social supports when they face difficulties, increasing stress levels and resulting in mental disorders.

Characteristics of socially prescribed perfectionists include striving to achieve excessive, uncontrollable, and unrealistic standards set by significant others [38]. These goals are often established by parents, friends, or teachers, and the external motivation can override intrinsic motivation, which impedes adolescents’ sense of self-autonomy. According to the self-discrepancy theory proposed by Higgins, the existence of disparities between one’s actual self and perceived societal expectations, also known as the “ought” self, creates psychological discomfort, which also can lead to the emergence of emotions associated with dejection and agitation [41]. A longitudinal study conducted over a 6-month period revealed that socially prescribed perfectionism was directly associated with adolescent depression over time, and the interaction between socially prescribed perfectionism and life stress predicted self-harm behaviors [28].

In conclusion, it can be observed that teenagers who have a predisposition towards perfectionism tend to have elevated expectations regarding their performance. Adolescents experience negative feelings and anxiety not only when they fail to achieve certain requirements but also throughout their pursuit of perfection, which ultimately hinders their mental well-being.

**Table 2:** Types of perfectionism and relations with mental health.

Types of Perfectionism	Author and Year	Characteristics	Relations with Mental Health
Self-oriented perfectionism	Levine et al. (2019) [10]	Excessively high internal standard. Self-criticism and self-blaming.	Positive association with anxiety and depression symptoms.
Other-oriented perfectionism	Stoeber (2014) [27]	Idealized expectations of others. Other-oriented criticism when others fail to meet perfection.	Harms interpersonal relationships. Linked to narcissism, Machiavellianism, and (subclinical) psychopathy.
Socially prescribed perfectionism	Higgins (1987) [41] O’Connor (2010) [28]	Excessively high external expectations. Lack of intrinsic motivation.	Creates emotional distress. Positive association with depression and self-harm behaviors.

## 4. Family Environment 

Reviewed papers are summarized in Table 3, involving ten cross-sectional and longitudinal studies regarding how family environment was related to adolescents’ mental health. Family functioning refers to the household environment and how family members connect and interact in and maintain relationships as well as how they make decisions and handle issues together [42,43]. It contains factors of family cohesion, parental involvement, parenting styles, and parents’ own mental health [44]. The dyadic interactions between parents and children, which last from the children’s birth to their maturity, are a prominent influence on adolescents’ development trajectory in terms of emotion, psychological functioning, and mental health. 

### 4.1. Adverse Childhood Experiences (ACEs)

Family provides an environment for children’s early development, which lays the foundation for their later physical and psychological health. Experiencing traumatic events, for example, domestic violence, child abuse, and frequent family conflicts, adversely shape and influence the biological mechanism and growth of a child, causing more fragile physical and mental capacity. Within such a family ecosystem, characterized by a lack of safety and warmth but marked by violence and disruption, children’s perception of communication, conflict resolution, coping strategies and self-expression become distorted because there is no appropriate standard or pattern to follow. A stress sensitization model was proposed to explain the diathesis–stress model [36]. The early exposure to adversities for children makes them more susceptible to developing depression later in adolescents when faced with the same amount of stress, effectively lowering the threshold for a depressive state [36]. Teenagers with numerous ACEs reported a 3 to 15 times greater likelihood of having various health events including cognitive difficulties, depressed mood, self-injurious behaviors, and suicidal ideation when several demographic variables were controlled [45]. 

### 4.2. Parent Status

Socioeconomic variables within families are related to adolescents’ psychiatric disorders. Analyzing the national data on US adolescents through logistic regression, a fully adjusted model demonstrated that parents’ education backgrounds and children’s perceived social status were associated with past-year psychological disorders [46]. A meta-analysis of 13 population-representative cohorts in the US since 1980, involving 26,715 participants aged 3–19, revealed small to moderate associations between various indicators of low socioeconomic status (SES) (e.g., family income, parental education, poverty status) and higher levels of childhood psychopathology [47]. Household income levels determine nutritional intake, living conditions, physical development, and the quality of education provided to children, all of which are closely related to individual well-being. Physical materials are directly related to the biological condition of a person, including brain development and the immune system. Rich education resources provide a path for healthy cognitive development, self-actualization, and goal attainment, which meet individual spiritual needs. A longitudinal study examined the relationship between poverty and mental health among a group of teenagers at ages 13 and 17 [48]. The results revealed that chronic childhood poverty predicted increased internalizing problems (anxious, depressed, and socially withdrawn) and externalizing problems (aggressive behaviors), with disengagement coping strategies serving as a mediator [48]. Furthermore, a survey study showed that family poverty is also associated with low self-esteem, mediated by deprived parental involvement and parents’ poor mental health status [49]. It could be perceived that numerous risk factors leading to parents’ poor mental health are directly or indirectly influenced by household capital.

**Table 3:** Summary of studies regarding family environment.

Area	Author and Year	Region	Target Age (yrs)	Sample Size	Measurements and Outcomes	Key Findings
ACEs	Meeker et al. (2021) [45]	US	adolescents	1532	Eleven items of stressful or traumatic situations. Measures of Health Risk Indicators. Measures of violence engagement and victimization.	Youth with multiple ACEs were more likely to experience varying cognitive difficulties and depressed mood. Multiple ACEs elevated the probability of suicidality, violence, and substance use.
Parent status	McLaughlin et al. (2012) [46]	US	13–17	904	Composite International Diagnostic Interview: psychological disorders. Parent education and family household income: Absolute Socioeconomic status. MacArthur SES and Health Network: Subjective social status.	Education attainment of parents was associated with child past-year psychological disorders. Child past-year disorders were linked to subjective social status.
Parent status	Kim et al. (2016) [48]	US	Initial test: 13 Follow up: 17	185	Income-to-needs ratio: Poverty exposure. Children’s Coping Strategies Checklist (CCSC). Response to Stress Questionnaire (RSQ). Adolescent Perceived Events Scale. The Youth Self-Report: Behavioral adjustment.	Poverty exposure from birth to age was chronologically associated with internalizing and externalizing symptoms. The longer the poverty exposure, the more likely adolescents were to adopt disengagement coping strategies which predicted internalizing and externalizing symptoms.
Parent status	Doi et al. (2019) [49]	Japan	4th grade 6th grade 8th grade	1652	Items about child poverty. Japanese version of the Children’s Perceived Competence Scale. Japanese version of the Kessler 6 (K6): Parental mental health. Items of parental involvement. Items of school social capital. Items of parental social capital.	Exposure to child poverty was associated with decreased self-esteem, mediated by poor parental involvement. Poor parental mental health and poor parental social capital acted as partial mediators in the link between child poverty and poor parental involvement. Poor school social capital mediated the association between poor parental involvement and low self-esteem.
Parenting styles	Kallay and Cheie (2023) [50]	North- West Romania	16–18	202	Adolescent Psychopathology Scale—Short Form. 40 items questionnaire of perceived parenting (emotional warmth, rejection, and control). The Cognitive Emotion Regulation Questionnaire.	Perceived parental warmth predicted children’s positive psychological adjustment and positive personality traits, mediated by positive cognitive emotion regulation (ER) strategies. Perceived parental rejection predicted children’s externalizing symptoms and social interaction disturbances, mediated by maladaptive ER strategies. Perceived parental control predicted children’s internalizing symptoms, mediated by maladaptive ER strategies.
Family connection	Ackard et al. (2006) [51]	US	7th–12th grades	4746	Items of Parent–Child Connectedness. Items of behavioral health: Substance use, suicide attempts. Rosenberg Self-Esteem Scale. Depression scale by Kandel and Davies.	Teenagers who reported low communication with their mother or father tended to report greater prevalence of health. Parent–child relationships that prioritized friends’ opinions over parents’ and limited communication about issues were linked to body dissatisfaction, low self-esteem, and depression.
Family connection	Keskin and Çam (2010) [52]	Turkey	11–16	384	The Strengths and Difficulties Questionnaire (SDQ): Strength and problematic behaviors. The Adolescent Relationship Scales Questionnaire (A-RSQ): Attachment styles.	Positive correlations between fearful attachment and emotional symptoms. Securely attached pattern was negatively related to emotional symptoms, hyperactivity–inattention, and peer problems. Dismissing attachment was positively correlated with emotional symptoms and hyperactivity–inattention and negatively correlated with social behaviors.
Family connection	Treleaven (2023) [53]	Mali	Under 5	3948	Characteristics of extended family: formal schooling, sole or shared decision-making power, and participating in any paid labor outside the home. Child health outcomes: any care and care from qualified providers.	The decision-making power of distant female family members had a favorable correlation with a child’s likelihood of receiving care from a qualified provider.
Family connection	Turney (2023) [54]	US	Longitudinal: From 1 to 9	4342	Perceived instrumental support. Children’s health ratings.	A favorable correlation between mothers’ views of instrumental assistance and the general health of their children was found.
Parental involvement	Wang and Sheikh-Khalil (2014) [55]	US	15–17	1056	Measure of parental involvement. Measure of academic engagement. Grade points: Academic achievement. Twenty items from the Children’s Depression Inventory.	Home-based involvement and academic socialization were positively linked to academic achievement. School-based involvement and academic socialization were negatively linked to depression. Behavioral and emotional engagement played a potential mediate role in the relationship between parental involvement and GPA and depression.

### 4.3. Parenting Styles

Parenting styles, classified by Baumrind [56], have been well validated by researchers and are defined as authoritarian, authoritative, and permissive. Both authoritative and authoritarian parents attempt to impose their self-defined standards and exert a certain degree of control over children’s activities. The main differences lies in the fact that authoritative parents provide a logical explanation for their order, while authoritarian parents reject the child’s desires and tend to resort to punishment [56]. The development of self-autonomy and the ability to initiate activities according to one’s own will are indispensable tasks during the development of an independent personality. Adolescents struggle to discover their intrinsic motivation under the high stress of obeying parent’s commands, and their sense of autonomy is repressed, leading to adverse mental states. Functional parenting provides necessary guidance and resources when needed, and stable emotional support from parents is also essential, as it helps contain and resolve children’s psychological distress. However, control-based parenting limits the autonomy and initiative of children, which hinders their psychological development. A meta-analysis covering studies over the past two decades indicates that parental psychological control is a significant risk factor contributing to an elevated likelihood of internalizing disorders in children, including depression and anxiety, with moderate effect size [57]. Substantial evidence has shown that the relationship between parental psychological control and children’s internalizing problems is universal, with a stronger impact in collectivist cultures [57]. Children under permissive parenting are granted a high degree of flexibility to pursue their own desires and face low parental expectations [56]. Although their need for autonomy is met, the lack of appropriate parental guidance and unfulfillment of responsibility can be harmful for children. Indulgent parenting creates an environment where children may perceive themselves as the center of the world, lacking the capacity for self-regulation, conflict resolution, and the formation and maintenance of social relationships. Children in permissively indifferent families may feel their needs are overlooked and become gradually emotionally isolated from their parents, which can damage their self-esteem and self-confidence. When children are confronted with difficulties, their distress is not buffered by parental support, leading to greater mental pain and fragility. 

Contrary to the malfunctioning parenting mentioned above, positive parenting greatly benefits children’s cognitive, emotional, and behavioral functioning. Previous research findings highlighted the critical role of parental engagement in providing care, emotional support, and consolation to their children. Kallay and Cheie [50] carried out a study aiming to investigate the association between adolescents’ perception of parenting practices and their symptomatology with a 40-item self-report questionnaire. The results indicated that the family practice providing sufficient emotional warmth was a predictor of increased well-being, parental rejection was predictive of externalizing and social interaction problems, and parental control was predictive of internalizing symptomatology [50]. Similarly, a meta-analysis demonstrated that perceived parental warmth had a substantial correlation with children’s psychological adjustment (e.g., positive self-esteem, self-adequacy, and well-being) and personality traits (e.g., less hostility, aggression) [58]. Given the pivotal role of parental practices in the development of children’s mental health, further study could focus on the mechanisms of parental warmth’s contribution to positive mental health outcomes in children.

### 4.4. Family Connection

A cohesive family connection is essential in rearing children. Packard and colleagues [51] conducted a large sample of population-based studies examining parent–child connectedness and behavioral and emotional health. The results revealed that perceived low maternal care for both boys and girls was significantly associated with maladaptive weight control and suicide attempts. Furthermore, mistrust in parental opinions was identified as a risk factor for negative emotional outcomes, including depression, body dissatisfaction, and low self-esteem [51]. Ainsworth classified attachment styles into secure, anxious–avoidant, and anxious–resistant [59]. A harmonious family atmosphere provides children with a sense of belonging and support, creating their own secure attachment. Secure attachment positively influences adolescents in various aspects, such as social skills, emotion regulation, and mental well-being. By internalizing the belief that support is always available, a securely attached individual develops robust psychological resources to deal with distress and makes it possible to decrease the adoption of psychological defense mechanisms that constrain coping options and cause conflict with others [60]. Research has found that emotional difficulties, attention deficits, hyperactivity, and peer difficulties are less problematic for adolescents who had developed secure attachment patterns while being more problematic for youth with dismissing attachment patterns [52]. The communication patterns between children and parents extend out of the family context to various social interactions with peers, friends, and co-workers in the future, interfering with the development of positive relationships later in life. If children feel loved and respected by family members, they are naturally more likely to exhibit these characteristics in social relationships and find a sense of belonging in society. 

Beyond the influences of the nuclear family, the connection between children and the extended family holds considerable importance. On the one hand, the extended family could compensate for the financial status of the nuclear family. On the other hand, a supportive extended family often enriches adolescents’ psychological resources that mitigate emotional distress and address difficulties. Notably, relationships with extended family benefit children’s health and well-being through providing direct or indirect support to mothers [53,54]. There is a positive association between mothers’ perceptions of instrumental support from extended family and children’s health [54]. The dynamics of negotiation between mothers and female extended relatives was manifested in the decision-making process [53]. It was found that the decision-making ability of extended female relatives predicted the quality of health care services received by children [53]. However, there is a gap in research regarding the influence of paternal relatives on the nuclear family. And further studies are necessary to delve into the more specific relationship between extended family and the psychological well-being of adolescents besides young children.

### 4.5. Parental Involvement

One of the primary routines for adolescents is attending school for education and preparation for future careers as adults. Active parental involvement in children’s academic goals is a sign of benign family interaction. Appropriate parental guidance on transition to adulthood and career direction helps adolescents understand the significance of their current striving in academic performance, which is an efficient type of parental involvement. Parental involvement encompasses home-based support, school-based engagement, and achievement socialization, which can manifest as behavioral actions (i.e., communication with school, homework assistance), intellectual support, cognitive guidance, and personal approaches [61]. Given that academic success is critical for mental health, previous research has explored the role of parental involvement in this process. The results indicated that academic socialization and school-based involvement have a direct positive impact on mental health and protect adolescents from depression by supporting children’s confidence and fostering opportunity for personal identity development [55]. Academic socialization involves communicating the value and practicality of education, connecting academic knowledge with social events, and establishing future occupational objectives, all of which fully prepare adolescents for the next stage in life [61]. Effective parental involvement helps alleviate adolescents’ stress and confusion about their current schoolwork, ultimately promoting better mental health.

## 5. Social Relationship

Sixteen cross-sectional and longitudinal studies are included in the discussion of the influence of social relationships on the development of psychological disorders, examining perspectives such as peer rejection, bullying, peer victimization, and the school climate (Table 4).

### 5.1. Peer Relationships 

Peer relationships involve dimensions of acceptance, intimacy establishment, and trustworthiness among peers, which are essential for adolescents’ psychological health [62]. A study conducted with a sample of German teenagers found that regression models showed that low levels of peer acceptance, dependability, and sociability both individually and collectively predicted more severe depressive symptoms [62]. Furthermore, a study reviewing 17 experimental studies indicated that peer rejection is closely linked to psychological distress, negative emotional effects, and depressive symptoms [63]. Consistently, adolescents with the poorest peer relationship, as well as a lack of school connectedness, reported the worst outcomes related to depression, anxiety, and overall well-being [64]. 

The opposite of peer acceptance is peer rejection, which manifests in forms of social isolation and victimization. Peer rejection and peer exclusion can cause great psychological distress in adolescents and are risk factors for psychological disorders. The psychobiological model suggests that peer rejection acts as an elicitor on brain regions related to distress, negative self-evaluation, and emotion [65]. Social rejection also strongly activates experiences of shame, which in turn trigger an inflammatory response [66]. The biological reaction, characterized by the release of pro-inflammatory cytokines accompanied by feelings of shame, subsequently leads to a range of behaviors associated with depression [65]. Given that these findings are preliminary, further research is essential to explore the biological processes ensuing from peer rejection. A research involving a sample of 511 teenagers aged 12–17 revealed that a higher level of peer rejection was related to a lower level of self-potency and a higher level of depression symptoms [67].

The primary pathway through which peer relationships affect mental health is social support. Adolescents with poor peer relationships have less social support, daily entertainment resources, and companionship, which are closely related to daily emotional states. A lack of social support has been linked to increased negative emotional states, as demonstrated by a study of a Chinese sample during the COVID-19 outbreak period, with rumination and sleep quality mediating this effect [68]. Prolonged exposure to negative states that elicit a stress response can contribute to depression and anxiety in adolescents. Furthermore, frequent rumination that is negatively related to social support is a prominent risk factor for depressive episodes [69]. Therefore, unsatisfactory peer relationships interfere with sources of social support, trigger negative cognitive styles like rumination, and ultimately elevate vulnerability of negative mood and even depressive disorders.

**Table 4:** Summary of studies regarding social relationships.

Area	Author and Year	Region	Target Age (yrs)	Sample Size	Measurements and Outcomes	Key Findings
Peer relationship	Adedeji et al. (2022) [62]	Germany	14–17	446	Patient-Reported Outcome Measurement Information System (PROMIS)—Pediatric peer relationship measure. Seven-Item version of the Center for Epidemiological Studies Short Depression Scale.	The severity of depressive symptoms was uniquely predicted by factors such as peer acceptance, friend dependability, and easiness to make new friends.
Peer relationship	Widnall, E., et al. (2022) [64]	UK	13–14	603	The 14-item Hospital Anxiety and Depression Scale (HADS). The 14-item Warwick and Edinburgh Mental Well-Being Scale (WEMWBS).	Levels of anxiety, depression, and well-being were associated with peer and school connectedness and were consistently poorest among students who were least connected to their school and peers before the COVID-19 pandemic. There was a dramatic decrease in anxiety during the lockdown for students who were least connected to school and peers and an increase when going back to school.
Peer relationship	Beeri and Lev-Wiesel (2012) [67]	Israel	12–17	511	Social Rejection Scale. PTSD scale. Beck Depression Inventory. Social avoidance and distress scale (SADi). Potency scale. Perceived social support—PSS.	Social rejection elevated psychological discomfort including PTS symptoms, depression symptoms, and social avoidance in teenagers. Social rejection lowered the level of personal resources
Peer relationship	Guo et al. (2022) [68]	China	12–18	1065	Chinese version of Depression Anxiety Stress Scale (DASS). Pittsburgh Sleep Quality Index (PSQI). The Ruminative Responses Scale (RRS). Social Support Rating Scale (SSRS).	The level of social support was predictive of the level of negative emotion, which was mediated by rumination and sleep quality.
Peer relationship	Robinson and Alloy (2003) [69]	US	College freshmen	170	The Response Styles Questionnaire (RSQ). The Stress-Reactive Rumination Scale (SRRS). Schedule for Affective Disorders and Schizophrenia—Lifetime (SADS-L). The Beck Depression Inventory (BDI).	Individuals with negative cognitive styles and ruminative tendencies after stressful events had higher rates and duration of major depression and “hopeless depression” episodes.
Peer relationship	Sollar et al. (2017) [70]	US	Adolescents	15,000	The Center for Epidemiologic Studies Depression Scale. Binary variables of sexual behavior. Seventeen ideal events in romantic relationships: inauthenticity.	Engaging in sexual intercourse was linked to an increase in depressive symptoms in both girls and boys. The correlation between sexual activity and females’ mental well-being was especially evident in partnerships marked by high degrees of inauthenticity.
Peer relationship	Exner-Cortens et al. (2013) [71]	US	12–18	5681	Audio computer-assisted self-interview about dating violence victimization. Twenty-item Centers for Epidemiologic Studies—Depression Scale. Rosenberg’s self-esteem scale. Self-Reported Delinquency scale. Dichotomous variable of suicidality. Dichotomous variable of substance use.	Dating violence was associated with depression symptomatology, suicidal ideation, and severe alcohol use among girls. Dating victimization was associated with increased risks of antisocial behaviors, severe alcohol drinking, and substance use among boys.
Victimization and bullying	Ringdal et al. (2020) [72]	Norway	15–21	1814	The Multidimensional Scale of Perceived Social Support (MSPSS). Norwegian Survey on Living Conditions 2012: Bullying. Ten-item Hopkins Symptom Checklist (HSCL-10). the Adolescent Stress Questionnaire (ASQ).	Friends and family support buffered anxiety and depression symptoms. The experience of being bullied was strongly associated with anxiety and depression symptoms.
Victimization and bullying	Chang et al. (2013) [73]	Taiwan	10th grade	2992	Items about cyberbullying and school bullying. Rosenberg self-esteem scale. Center for Epidemiologic Studies. Depression Scale (CES-D).	School victims and bullying victims were correlated to lower self-esteem and higher depression.
Victimization and bullying	Thomas et al. (2016) [74]	Indonesia	Majority 15 or under	10,883	Items about suicidal behaviors. Items about psychological distress. Items about experience of being bullied.	Psychological distress, for example, loneliness and sleep disturbance, mediated the association between bullying victimization and suicidal behaviors.
Victimization and bullying	Zimmer-Gembeck, et al. (2014) [75]	Australia	10–14	366	Children’s Rejection Sensitivity Questionnaire (CRSQ). Children’s Depression Inventory (CDI). The Children’s Social Behavior Scale: Peer victimization. Three items of friendship conflicts.	Victimization was associated positively with rejection sensitivity and negatively associated with depressive symptoms and loneliness. Rejection sensitivity moderated the association between friendship conflict and adjustment ability (loneliness and depressive symptoms).
Victimization and bullying	Nepon et al. (2021) [76]	Canada	*M_age_* = 15.2	1039	Bullying questionnaire. Rejection Sensitivity Questionnaire. Self-description questionnaire I: Self-esteem. Reynolds Adolescent Depression Scale.	Rejection sensitivity and self-esteem were identified as potential mediators of the longitudinal associations between peer victimization and both depressive symptoms and substance use. Peer victimization was correlated to lower self-esteem, higher rejection sensitivity, and more mental health problems.
School climate	La Salle et al. (2021) [77]	14 countries and regions	11–17	34,923	The Georgia School Climate Survey (GSCS). Georgia Student Health Survey 2.0.	In majority groups, school climate was associated with mental health outcomes.
School climate	Franco (2022) [78]	US	12–14	2768	Classroom Climate Scale. Generalized Anxiety Disorder. The Center for Epidemiologic Studies Short Depression Scale. Symptom Checklist-90: Hostility. Impulsiveness Subscale from the Teen Conflict Survey.	Positive relationships with peers were associated with less depression. Relationships with teachers were positively associated with anxiety symptoms. Awareness of support resources and seeking help for negative events was related to less anxiety, depression, hostility, and impulsivity.
School climate	Yang et al. (2019) [79]	US	Parents of 4th–12th graders	11,484	Delaware Bullying Victimization Scale—Home: Bullying Victimization, Teacher–Home Communication, and Fairness of Rules.	Teacher–home communication was associated with perceived less frequent bullying victimization, moderated by perceived fairness of rules. The magnitude of the association between teacher–home communication and bullying victimization was greater in schools with less fair rules.
School climate	Varela (2021) [80]	Santiago de Chile	9–16	366	The school climate scale. Illinois Bullying Scale. Pediatric Symptom Checklist for Adolescents: Internalizing and externalizing behaviors.	Positive school climate was a predictor of fewer school bullying events. Positive school climate was predictive of fewer internalization problems both directly and indirectly through reducing the frequency of being a victim of bullying.

A special form of peer relationships among adolescents, romantic relationships, need attentions. Adverse impacts on mental health might be brought on by the break-up of romantic relationships that are more serious among adolescents than adults. Another problem accompanying romantic relationships is early sexual intercourse, which is a risk factor for emotional health. It was discovered that the negative impact of sexual activity within romantic relationships on mental well-being (i.e., depression) was most noticeable in girls who had high levels of relationship inauthenticity [70]. Similarly, for boys, experiencing sexual intercourse was associated with higher risks of depression [70]. Dating violence is also a concern for adolescents. A longitudinal study showed that teen dating violence could result in serious mental disorders, including depression symptomatology, suicidal ideation, and severe alcohol use among girls five years after the event [71]. For boys, teen dating victimization was also associated with increased risks of antisocial behaviors, severe alcohol drinking, and substance use [71]. These research findings suggest the need for sexual education and victimization prevention programs for adolescents to mitigate the potential harmful impact of teen romantic relationships.

### 5.2. Victimization and Bullying 

Extensive research has demonstrated the fatal impacts of bullying on adolescents’ mental health. Being bullied was negatively associated with adolescents’ well-being and positively associated with depression and anxiety symptoms [72]. When gender, academic performance, and household income were controlled, adolescents who had experienced bullying were more likely to have lower self-esteem and were more vulnerable to serious depression [73]. A study revealed that all forms of bullying including verbal, relational, and physical bullying elicited strong psychological distress and diminished emotional well-being [74]. When victimization occurs in the school setting and, at the same time, students cannot avoid staying on campus, victims become embroiled in prolonged distress that is brought about by the victimization. If this distress cannot be discovered by teachers or parents and addressed effectively, the continuous negative mental state can lead to various psychological disorders.

Prolonged exposure to victimization or bullying has damaging impacts on adolescents which lasts throughout adulthood. Longitudinal studies have found that peer victimization is linked to depressive symptoms, substance use, loneliness, and emotional maladjustment, mediated by increased rejection sensitivity and reduced self-esteem [75,76]. Rejection sensitivity refers to an individual’s dispositional oversensitivity to social rejection [81]. Based on a social cognition model, Levy proposed that the defensive expectation of rejection is formed by a repeatedly unmet need for belonging to family and friends [82]. Consequently, the oversensitivity to imaginary rejection may result in actual rejection as a self-fulfilling prophecy because a person with high rejection sensitivity tends to perceive non-malicious clues as signs of rejection [83]. 

High sensitivity to rejection means an excessive concern for external evaluation without a stable inner standard and a clear self-view. For adolescents, peer acceptance and recognition are vital sources of a sense of self-worth, belongingness, and social connection that are closely related to healthy psychological development. While peer victimization strengthens the response to rejective signals in social interactions, it becomes challenging to develop a positive self-view. Instead, adolescents frequently feel disapproved of and are prone to depression.

### 5.3. School Climate

There is a growing tendency to focus on the influence of school climate on teenagers’ behavioral and psychological health as well as their overall well-being. Aside from family, school is a place where adolescents spend a significant amount of time on education. Definitions of school climate may differ among studies, educators, and researchers. There is a general consensus that school climate refers to the comprehensive and multi-faceted assessment of the social environment within a school [84]. School climate encompasses various aspects of school life, such as safety, relationships (e.g., diversity, connectedness), teaching and learning, and the school environment (e.g., material and places) [18]. It also includes broader organizational patterns, ranging from fragmented to cohesive or having a “shared” vision, and can be characterized as healthy or unhealthy, conscious, or unrecognized. A systematic review reported that 46 out of 48 reviewed studies found that students’ favorable perceptions of school policies and regulations, cohesive relationships between peers, and a sense of belongingness were associated with elevated prosocial behaviors, psychosocial well-being, reductions in mental ill-health, and decreased risk behaviors [16]. School policies and their implementation serve the function of directing expected and correcting improper behaviors of students. Subsequently, in a study of 34,923 secondary school students across schools in 14 territories, evidence suggested the positive functions of a supportive school climate for mental health, which was measured in aspects of symptoms of depression or emotional dysregulation [77]. The results demonstrated the school climate’s eight dimensions (school connectedness, character, physical environment, adult social support, peer social support, cultural acceptance, order, and discipline and safety) were collectively associated with mental health in nine regions [77]. 

Furthermore, school climate contributes to healthy psychological well-being through indirect effects, as it promotes amiable peer relationships and prevents malignant events such as bullying. In a sample of 2768 middle school students in the US, better inter-student relationships were associated with reduced externalizing problems and less depressive symptoms, while stronger student–teacher relationships predicted less hostility but an increase in anxiety [78]. A possible explanation for the inverse relationship between student–teacher relationships is that students who have higher levels of anxiety may inherently seek more support from teachers and, thus, form a positive perception of student–teacher relationships [78]. The awareness of reporting victimization and trust in teachers’ capacity to resolve victimization have the strongest association with positive outcome of students’ levels of hostility, impulsivity anxiety, and depression symptoms [78]. 

Not only is school climate an issue between students and teachers, but it is also important to note that the promotion of parent–school communication and collaboration is beneficial to constructing a better school climate. Research has provided empirical evidence supporting the negative association between parent–teacher communication and school bullying [79]. The strength of this association was found to intensify from elementary to high school and was more pronounced in schools with less equitable school rules [79]. A reduction in victimization and bullying is of huge importance in protecting adolescents from developing mental disorders. A study conducted in Santiago de Chile among 9–16 year-old adolescents indicated that a positive school climate predicted less occurrences of victimization and consequently lower levels of internalizing and externalizing problems [80]. In a low-connection climate, bullying is less likely to be intervened upon by peers, as indifference in social conducts abets by-standers and perpetrators. At the same time, ineffective actions by teachers to punish improper behaviors harm the trust of students. Students who have low trust in school officials are less prone to seek external help. For students in vulnerable positions, lacking accessible resources to resolve victimization or recovering from physical and psychological harm can lead to increasingly serious mental problems. Therefore, it stands to reason that a negative school climate can lead to an outburst of mental health problems. 

## 6. Discussion

Mental health problems among teenagers have been receiving increasing attention, and there is a growing tendency for youth to suffer from depression, anxiety, and decreased well-being. Puberty, a critical developmental stage marked by physical, emotional, and cognitive changes, renders adolescents more vulnerable to mental disorders. Therefore, it is essential to identify factors that are linked to the vulnerability of mental illness in teenagers. The current study aims to shed light on how risk factors at three levels, including individual vulnerability, family environment, and social relationships, impact adolescent mental disorders: for example, depression, anxiety, and suicidal thoughts and attempts. 

**Figure 1:**
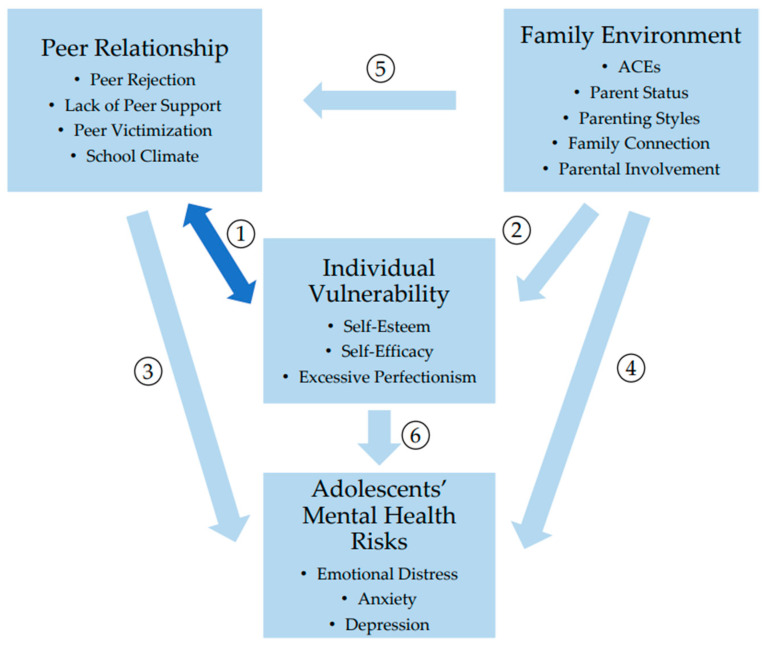
Summary of the interaction between individual vulnerability, family environment, peer relationships and adolescents’ mental health. ① Individual vulnerability is bidirectionally predictive of peer relationships. ② Family environment is linked to individual vulnerability. ③ Peer relationships are associated with adolescents’ mental health issues. ④ Family environment is associated with adolescents’ mental health issues. ⑤ Family environment influences peer relationships. ⑥ Individual vulnerability is associated with adolescents’ mental health issues.

Nevertheless, the three layers of individual vulnerability, family environment, and social relationships are not mutually exclusive but intrinsically connected as shown in Figure 1. It is essential to note that family practices and peer relationships more often participate in shaping personality, forming coping strategies, and shaping cognitive styles as indirect factors. For example, peer rejection and peer victimization are hazardous to social efficacy and self-esteem, as these experiences give rise to the sense of uncontrollability and failure in social interactions. An insecure family environment that fails to provide sufficient material and emotional support also undermines children’s self-worth and perceived controllability of life, which serves as an elemental factor associated with the development of psychological diseases. Eventually, negative self-impression and negative perception of the world increase the likelihood of mental malfunctioning because they present a universal rejection of one’s capabilities. Conversely, perfectionism could also deteriorate peer relationships and is significantly associated with bullying and social hopelessness [85]. In addition, while trait perfectionism is a risk factor to mental problems, parenting tremendously cultivates children’s personalities. The tendency toward perfectionism may be passed down to a child in the process of parental involvement when parents set an excessively high standard for children’s academic achievement. Investigators have demonstrated the predictive role of maternal perfectionism and psychopathology on child perfectionism [86]. Furthermore, a meta-analysis concluded that authoritarian and neglectful parenting are related to lower self-esteem in offspring [87], which makes them more vulnerable to psychological problems. The bidirectional interactions between individual factors and social factors as well as family practices jointly form the vicious spiral contributing to the development of psychological disorders. A review study suggested that parental psychological control and child maltreatment are two significant risk factors for peer victimization by indirectly hindering the development of appropriate self-cognition including self-efficacy and self-esteem [88]. It is advisable for future research to involve mediation and moderation analysis among those factors mentioned above.

The rapid development of technology in recent decades has given rise to concerns about social media use and its influence on mental problems. Studies involving adolescent samples have produced evidence of a positive association between heavy social media use (i.e., time spent on social media) and psychological distress, suicidal ideation [89], depression, and anxiety [90]. Future studies are needed to investigate whether different types of social media use and applications of social media have distinct impacts on the health concerns of youths. More importantly, it is crucial to identify effective strategies implemented within family and school environments to prevent or mitigate the negative effects of social media use. The potential adverse impact of romantic relationships on adolescents’ mental health outcomes, the dating experience on the online dating platform, and possible influences are also worth investigating in future research. Furthermore, it is worth noting that adolescents may be susceptible to psychological problems because of stressful life events, such as parental divorce. These events can trigger acute stress in a very short timeframe. A longitudinal study with a group of teenagers aged 11–15 suggested stressful life events in four domains (family and parents, romantic relationships, school and classes, and friends and social activities) are predictors of youth anxiety and depression symptoms [91]. 

Sex difference is an important dimension in the study of adolescents’ mental health development. Extensive research has examined the systematic differences in the prevalence and trajectory of psychological disorders between men and women. The National Epidemiologic Survey in the US revealed that women exhibit higher rates of mood disorders and anxiety, while men are more frequently diagnosed with substance use disorder and antisocial disorder [92]. Although the risk factors discussed in this study generally apply to both sexes, as most of the studies include participants of both genders, sex differences could still be a concern. It has been demonstrated that gender moderates the negative impact of anxiety and depression symptoms on various mental health outcomes. [93]. Specifically, boys are more adversely affected in areas such as self-esteem, academic problems, psychosocial functioning, and subjective well-being compared to girls [93]. Moreover, the same risk factor might also trigger different symptoms across genders. A study of 868 teenagers found that boys with a higher number of adverse childhood experiences (ACEs) are more likely to exhibit increased levels of externalizing behaviors [94]. 

Furthermore, the gender difference also contributes to negative outcomes via distinct pathways. A large-scale longitudinal study in the Netherlands discovered gender differences in the association between friendship quality and well-being [95]. For boys, high-quality friendships were directly linked to well-being and positive self-esteem [95]. In contrast, for girls, high-quality friendships did not directly influence their well-being in the long term but exerted an indirect effect through the enhancement of global self-esteem [95]. This discrepancy may be attributed to girls’ co-rumination tendency in a dyadic relationship, leading to the revisitation of negative experiences and emotions, especially when girls experience greater emphatic distress than boys during the process [96]. Gender differences also play a role in the reaction of the hypothalamic–pituitary–adrenal (HPA) axis to peer victimization [97]. A study involving 150 Chinese students aged 9–13 found that relational peer victimization was only associated with blunted cortisol reactivity among boys, not girls [97]. For boys, through the pathway of cortisol reactivity, peer victimization was indirectly linked to internalizing and externalizing problems [97].

The current study provides insights for relevant interventions and prevention programs. This study suggested a potential direction for cognitive behavioral therapy that focuses on a positive cognitive style through which individuals perceive and evaluate themselves, form expectations of others, and set goals. Programs aimed at promoting parent–child relationships and educating parents about child-rearing practices would be helpful for children’s mental health development. In addition, schools take a vital role in implementing regular mental health promotion training for teachers, which enables them to effectively recognize potential symptoms, particularly with regards to the emotional well-being of students following traumatic incidents occurring within the school environment.

## 7. Limitation and Future Research

This study mainly emphasizes the risk factors associated with common mental disorders focusing on individual characteristics, family practices, and social relationships. However, this approach is not exhaustive. It is believed that a constellation of factors, including family heredity, prenatal conditions, ethnicity, religions, community atmosphere, and culture influences, collectively impact psychological health. 

Although the reviewed studies provide substantial evidence of how risk factors from various contexts can adversely affect the mental health development of teenagers, most only examine correlations between factors and mental disorders, without demonstration of causal relationship. In addition, the methods adopted in all these correlational studies are exclusively self-report measurements. While the self-report scales used are generally well validated, future research would benefit from incorporating more diverse methods, such as evaluations by significant others and behavioral tasks, to effectively minimize bias inherent in self-reports. Furthermore, the majority of studies were conducted in developed countries and regions, such as the US, Britain, and Canada. This focus might lead to relatively unified characteristics of samples in terms of household income, education background, society welfare system, and mental health service rates. The monotonous sample could lead to bias in the results. Therefore, further research is necessary to investigate risk factors that elucidate psychological disorders in adolescents from developing countries and cities, enhancing the understanding and intervention strategies across diverse global contexts.

## 8. Conclusions

A total of 37 experimental studies regarding the mental health of adolescents have been reviewed in this study. These studies are categorized into three main areas: individual vulnerabilities, family environment, and social relationships. Unfavorable factors such as low self-esteem, low self-efficacy, and high trait perfectionism detrimentally affect the mental development of youths. These factors are linked to either a distorted self-concept or unrealistic expectations. Adolescents who grow up under negative family practices, including ACEs, adverse economic status, parental psychological control, and lack of family connectivity, face increased risks of depression, anxiety, and behavioral problems. Moreover, peer relationships play a critical role in adolescent mental health. Extreme events, such as peer victimization, are likely to have a damaging impact, leading to emotional malfunctioning, substance use, and internalizing disorders. The school climate emerges as a crucial construct influencing mental health that has great implications for educators and teachers. For future research, it is essential to adopt diverse measures to assess psychological health to explore broader perspectives, including the impact of ethnicity, cultural backgrounds, and social influences on adolescent mental health.

## Figures and Tables

**Table 1:** Summary of studies regarding individual vulnerabilities.

Area	Author and Year	Region	Target Age (yrs)	Sample Size	Measurements and Outcomes	Key Findings
Self-esteem	Moksnes and Reidunsdatter (2019) [9]	Norway	15–21	351	Warwick–Edinburgh Mental Well-Being Scale. Hopkins Symptom Checklist: Depression and Anxiety. Rosenberg Self-Esteem Scale. Adolescent Stress Questionnaire.	Self-esteem was predictive of depression and anxiety. Family economy was associated with well-being.
Self-esteem	Liu et al. (2021) [21]	China	10.9–15.4	1256	Rosenberg Self-esteem Scale. Mental Health Inventory of Middle School Students: for example, depression, anxiety, study stress, maladjustment, emotional instability, obsessive–compulsive symptoms, and paranoia. Social support rating scale. Ego-Resiliency Scale.	Self-esteem negatively predicted common mental health problems (CMHPs). Self-esteem mediated the association between social support and CMHPs. Self-esteem mediated the association between resilience and CMHPs.
Self-efficacy	Ahmad et al. (2014) [22]	Pakistan	16–19	216	Perceived Social Self-Efficacy (PSSE) scale: confidence in various social situations. Siddiqui–Shah depression scale.	PSSE was negatively associated with depression.
Self-efficacy	Cattelino et al. (2021) [23]	Italy	14–18	1004	Beck Depression Inventory-II. Grades in subjects: Social Achievement. Children’s Perceived Self-Efficacy. Single item of peer relationship.	Self-efficacy for self-regulation learning mediated the association between academic achievement and depressive symptoms. Peer relationships moderated the influence of self-efficacy on depressive level.
Self-efficacy	Schönfeld et al. (2016) [24]	Germany Russia China	Germany (*M_age_* = 26.33). Russia (*M_age_* = 21.39). China (*M_age_* = 21.5).	Germany (*n* = 394). Russia (*n* = 604). China (*n* = 8669).	Depression Anxiety Stress Scales (DASS-21). The Positive Mental Health Scale (PMH): Emotional, psychological, and social well-being (BDSS). The Brief Daily Stressor Screening (GSE).	Self-efficacy mediated the association between daily stressors and mental well-being
Self-efficacy	Cherewick et al. (2023) [25]	Tanzania	9–12	579	Self-Efficacy Questionnaire for Children (SEQ-C). The African Youth Psychological Assessment (AYPA): Internalizing and externalizing problems. Dweck’s Theories of Intelligence Scale: Growth mindset. Persistence Scale for Children.	Self-efficacy was a mediator between the protective association between motivation mindsets on children’s psychopathology. Higher domain specific self-efficacy in learning was associated with reduced externalizing problems.
Self-efficacy	Muris (2002) [8]	Belgium	12–19	596	Self-Efficacy Questionnaire for Children (SEQ-C): Social self-efficacy, academic self-efficacy, and emotional self-efficacy. State-Trait Anxiety Inventory for Children (STAIC). Screen for Child Anxiety-Related Emotional Disorders (SCARED). Children’s Depression Inventory (CDI).	Self-efficacy was significantly related to anxiety symptoms and depression. Social self-efficacy was connected to social phobia. Academic self-efficacy was connected to school phobia. Emotional self-efficacy is connected to panic/somatic and generalized anxiety.
Perfectionism	Levine et al. (2019) [10]	Canada	12–17	174	The Multidimensional Perfectionism Scale. The Brief Screen for Depression (BSD). The Costello–Comrey Anxiety Scale (C-CAS).	Self-critical perfectionism was positively correlated to anxiety and depression.
Perfectionism	Levine et al. (2023) [26]	Canada	*M_age_* = 17.98	658	Depressive Experiences Scale–Self-criticism Six-Item Scale (DEQ-SC6). Frost Multidimensional Perfectionism Scale (Frost-MPS). Revised Almost Perfect Scale (Revised-APS). The Center for Epidemiological Studies Depression Scale Revised (CESD-R). The Brief Symptom Inventory (BSI).	There was a positive correlation between self-critical perfectionism and anxiety and depressive symptoms throughout the school year. Perfectionism served as both a risk factor for increasingly severe levels of anxiety and depression symptom trajectories and also for less severe symptom trajectories.
Perfectionism	Stoeber (2014) [27]	Britain	17–50	Study 1: 338 Study 2: 326	Forty-five-item MPS and eight-item “1990” scale: Perfectionism. Items for social goals. Twelve-item Dirty Dozen scale. HEXACO Personality Inventory—Revised (HEXACO-PI-R).	Other-oriented perfectionism was inversely associated with nurturance and intimacy goals. Other-oriented perfectionism was positively associated with narcissism, Machiavellianism, and psychopathy. Other-oriented perfectionism measured by MPS was negatively associated with agreeableness. Other-oriented perfectionism measured by “1990” scales was negatively associated with emotionality and altruism.
Perfectionism	O’Connor et al. (2010) [28]	Scotland	*M_age_* = 15.2	737	Measure of 20 potentially stressful life events. Hospital Anxiety and Depression Scale (HADS). Single self-harm question. Fourteen-item Child and Adolescent Perfectionism Scale (CAPS-14).	Social-oriented perfectionism predicted increased later depression without moderation by acute life stress. Social-oriented perfectionism predicted increased later anxiety. Acute life stress predicted psychological distress.

## Data Availability

No new data were created or analyzed in this study. Data sharing is not applicable to this article.
